# Langerhans Cell Histiocytosis in Bilateral Mastoid Cavity

**DOI:** 10.1155/2013/957926

**Published:** 2013-06-11

**Authors:** Kazım Bozdemir, Behçet Tarlak, Hasan Çakar, Ahmet Doblan, Ahmet Kutluhan, İmdat Dilek, Nuran Adıyaman Süngü

**Affiliations:** ^1^Ankara Ataturk Training and Research Hospital ENT, Department Bilkent, 06800 Ankara, Turkey; ^2^Şehitkamil State Hospital ENT Department, 27500 Gaziantep, Turkey; ^3^Ankara Ataturk Training and Research Hospital, Hematology Department Bilkent, 06800 Ankara, Turkey; ^4^Ankara Ataturk Training and Research Hospital, Pathology Department Bilkent, 06800 Ankara, Turkey

## Abstract

A 39-year-old male was admitted to our clinic with symptoms of headache, dizziness, nausea, otalgia, otorrhea, tinnitus, and hearing loss in both ears for 3 weeks. Physical examination revealed edema in the tympanic membrane and external ear canal, and pain by palpation in the mastoid area bilaterally. There was no nystagmus, and the rest of the physical examination was otherwise normal. Temporal bone high resolution computed tomography (CT) showed a lesion causing erosion in the mastoid cortex, tegmen tympani, ossicles, and in the bone covering the sigmoid sinus bilaterally. There was also erosion in the superior semicircular canal and petrous bone on the left side. Cortical mastoidectomy was performed under general anesthesia. Histopathologic examination of the tissue revealed Langerhans cell histiocytosis (LCH). In this paper a case with LCH, presenting with bilateral mastoid involvement which has been rarely reported in the literature, is discussed with the existing literature.

## 1. Introduction

Langerhans cell histiocytosis (LCH) which originates from immature Langerhans cells is a clonal myeloproliferative disease with a variety of clinical presentations and prognosis. Although the lesions can be asymptomatic, it can present with swelling, pain, or pathological fractures in the site of involvement. In addition, there may be fever, loss of apatite, recurrent upper respiratory infections, cervical lymphadenopathy, otitis media, vertigo, facial paralysis, hearing loss, or hepatosplenomegaly. Mastoid bone involvement is not uncommon as well. Otologic involvement can be seen in 15% to 61% of the patients [1]. Although spontaneous recovery is possible in some cases, local curettage with or without postoperative radiotherapy is usually the choice of treatment. However, treatment of LCH includes surgical excision, radiotherapy, chemotherapy, and systemic corticosteroids either alone or indifferent combinations. Herein, we presented a case of LCH of the mastoid bone in an adult patient with bilateral aural involvement.

## 2. Case Report

A 39-year-old man was admitted with headache, dizziness, nausea, and otalgia, otorrhea, tinnitus, and hearing loss in both ears for 3 weeks. On physical examination, there was edema in the tympanic membrane and external ear canal as well as pain by palpation in the mastoid area bilaterally. There was no nystagmus. The physical examination was otherwise normal. Complete blood count, blood biochemistry, and serology were unremarkable. Tympanometry revealed type B tympanogram and absence of stapes reflexes on both sides. On audiometry, the pure tone averages were 17 dB and 28 dB on the right and left ear, respectively. There was a mild conductive hearing loss on the left ear. Video electronystagmography did not reveal a vestibular pathology.

**Figure 1:**
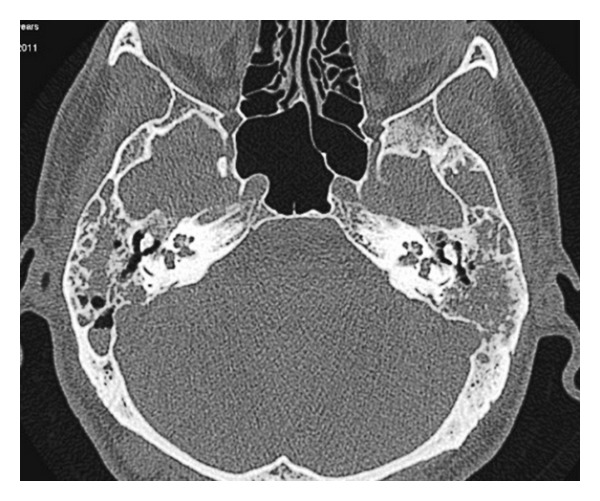
Preoperatively there is fluid intensity in the tympanic cavity and mastoid air cells. Note the irregularity in the bone covering the sigmoid sinus.

**Figure 2:**
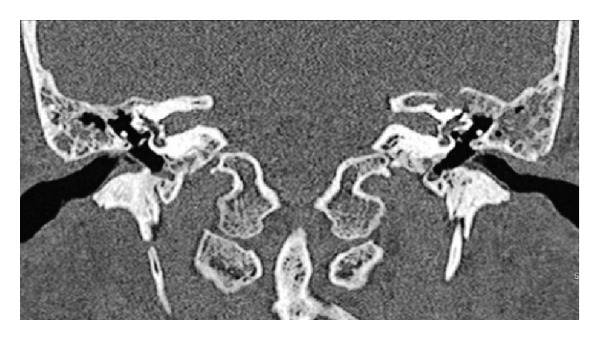
Note the erosion in the bone covering the superior semicircular canal and arcuate eminence as well as petrous bone.

High resolution computed tomography of the temporal bone showed a lesion causing erosion in the mastoid cortex, tegmen tympani, ossicles, and in the bone covering the sigmoid sinus bilaterally. There was also erosion in the superior semicircular canal and petrous bone on the left side (Figures 1 and 2). The intracranial structures were normal.

**Figure 3:**
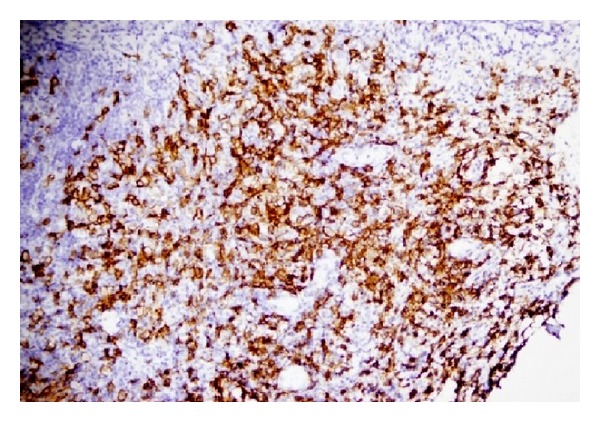
Histopathological assessment. Groups of cells with irregular and notched nuclei and acidophilic cytoplasm.

With a presumptive diagnosis of acute coalescent mastoiditis, an empiric systemic antibiotic (Ceftriaxone Sodium 1 g IV in divided doses twice a day) and topical ear drops (dexamethasone and ciprofloxacin) were started. After five days, a cortical mastoidectomy was made in the left ear. There was a hemorrhagic mass in the mastoid antrum. Histopathologic examination of this tissue proved LCH. Therefore, a cortical mastoidectomy was also made in the contralateral ear, and histopathology also proved LCH in this ear (Figure 3). A substantial recovery was observed in the symptoms of the patient in the early postoperative period. Otalgia, edema in the ear canal, and dizziness subsided.

In an attempt to disclose multifocal and systemic involvement, blood tests (peripheral blood smear, biochemistry, monoclonal gammopathy assay, sedimentation, C reactive protein) computed tomography of the thorax, whole body positron emission tomography, abdominal ultrasound, bone marrow aspiration biopsy, plain X-ray graphs of the long bones, and respiratory function tests were performed, and the results were unremarkable.

**Figure 4:**
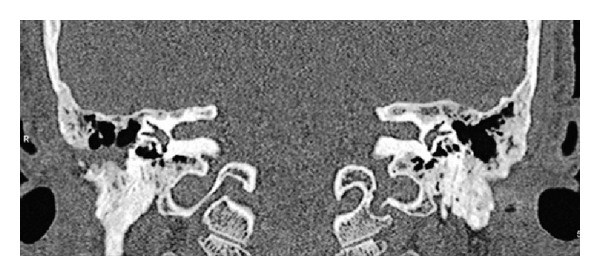
Postoperative CT scan. Note the absence of lytic lesion in left petrous apex and superior semicircular canal.

Three weeks after the last operation, under local anesthesia, the ears were injected with 4 mL (160 mg) of methyl-prednisolone acetate as advised by the hematologists. The temporal bone computed tomography which was obtained six months after the operation showed that both ears were free of the disease (Figure 4). The patient did well seven months after discharge.

## 3. Discussion

In the skull, LCH usually causes lesions in the frontal, parietal, and temporal bones. In a series of 314 patients with LCH, Howarth et al., skull involvement in 60% of the patients while the involvement of femur, pelvis, and vertebra was rare [2]. The clinical presentation of temporal bone involvement in LCH is highly variable such as otitis media, otitis externa, otorrhea, otalgia, postauricular skin rashes, and sudden hearing loss [3]. Among 24 patients with head and neck LCH, Anonsen and Donaldson reported mastoid bone involvement in 4 and petrous bone involvement in 1 of the patients [4].

There is no specific radiological finding for LCH. An osteolytic lesion and soft tissue intensities can be seen on computed tomography and magnetic resonance imaging. The diagnosis is made according to histopathological evaluation which shows histiocytes with eosinophilia in the cytoplasm and giant nucleus. The histiocytes are CD1a and S100 positive on immunohistochemistry.

In the treatment of LCH, surgery, chemotherapy, and radiotherapy are used in different combinations or a single modality. The treatment is individualized according to extension of the disease. Although low dose radiation (5–25 Gy) is an effective treatment, it is not recommended in the presence of the lesions which cause organ dysfunction by pressure [2–6]. Chemotherapy is recommended in the multifocal disease. Local corticosteroid application can be an effective treatment in the presence of a localized lesion [6]. Howarth et al. reported a spontaneous recovery in 3 of 188 cases with osseous LCH [2].

In conclusion, although LCH has been considered a systemic disease, isolated temporal bone involvement is also possible in which a misdiagnosis of otitis media or externa can be made. Isolated bilateral mastoid involvement should be even rare in LCH. Therefore, a high index of suspicion is required, and histopathological examination must be performed to justify the diagnosis.
